# Understanding the implementation and efficacy of a home-based strength and balance fall prevention intervention in people aged 50 years or over with vision impairment: a process evaluation protocol

**DOI:** 10.1186/s12913-018-3304-6

**Published:** 2018-07-03

**Authors:** Lisa Dillon, Lindy Clemson, Kristy Coxon, Lisa Keay

**Affiliations:** 1 Injury Division, The George Institute for Global Health, UNSW Sydney, Sydney, Australia; 2 Guide Dogs NSW/ACT, Sydney, Australia; 3 Faculty of Health Sciences, University of Sydney, Sydney, Australia; 4 School of Science and Health, Western Sydney University, Great Western Highway, Werrington, Penrith, NSW 2747 Australia

**Keywords:** Process evaluation, Strength and balance, Vision impairment, Falls prevention, Implementation

## Abstract

**Background:**

A nested process evaluation, within a randomised controlled trial, will explore relationships between program outcomes and quality of intervention implementation of the Lifestyle-Integrated Functional Exercise Program in older people with vision impairment. The Lifestyle-Integrated Functional Exercise Program is a home-based strength and balance program that has been shown to reduce falls in high risk populations. A pilot study showed positive trends in improvements in physical function in older people with vision impairment after participation in the program. The program will be delivered by Orientation and Mobility Specialists, who are experienced in working with people with vision impairment.

**Methods:**

The process evaluation has a mixed methods design. This includes quantitative (fidelity checklist score, number of completed sessions, survey data and a habit formation scale), as well as qualitative (open responses from program staff and semi-structured interviews with study participants) data. Process evaluation measures include program adherence (fidelity), complete delivery (dose delivered), participant receipt (dose received) and participant enactment. Using the Behaviour Change Wheel, a logic model was built to explain the intended inputs, outputs, outcomes and relationships to the behaviour change techniques in the Lifestyle-Integrated Functional Exercise Program in older people with vision impairment.

**Discussion:**

The findings of the process evaluation will inform the provision of fall prevention programs in older people with vision impairment by Orientation and Mobility Specialists. To date, there are no proven falls prevention programs which aim to improve physical function and reduce falls in older people with vision impairment. This process evaluation will contribute new knowledge about the implementation of a strength and balance program in this population.

**Trial registration:**

ACTRN12616001186448. Registered 29 August 2016.

**Electronic supplementary material:**

The online version of this article (10.1186/s12913-018-3304-6) contains supplementary material, which is available to authorized users.

## Background

Older people with vision impairment are eight times more likely to fall than their sighted peers [1]. With an ever increasing population of older people with vision impairment [2], it is crucial to carefully evaluate large scale fall prevention interventions that target this population. A 2012 Cochrane Review concluded that home and group-based exercise programs, along with home safety and medical management, reduce the rate and risk of falling in older people [3]. A recent systematic review by Sherrington et al. [4] identified that exercise as a single intervention can reduce falls by 21% in community dwelling older adults, particularly those interventions that include a high challenge to balance. Although programs which challenge strength and balance, such as the Otago Exercise Programme, have been found to reduce falls by up to 35% in the general population [5], one trial found no significant reduction in fall risk of participants with vision impairment [6].

The Lifestyle Integrated Function Exercise (LiFE) program [7] has been adapted for older people with vision impairment (v-LiFE) and evaluated in a pilot study. The v-LiFE program was delivered by Orientation and Mobility (O&M) Specialists, who have extensive experience delivering programs which increase independence and safety of those with vision impairment. A statistically significant reduction in fear of falling and improved late life function, as well as non-significant trends towards improvements in physical outcomes were reported, following participation in the program. Most participants were able to complete the program, and appreciated its benefits. It was concluded that O&M Specialists were well suited to delivering the v-LiFE program to this population. The pilot study did not have a control group or directly measure falls, but does provide support for a larger scale trial [8].

A larger scale single-blinded randomised controlled trial known as the PrevenTing Falls in a high risk, vision impaired population through specialist ORientation and Mobility services (PlaTFORM) commenced in 2017. A comprehensive protocol of the PlaTFORM study has been published elsewhere [9]. In brief, the aim of PlaTFORM is to investigate whether v-LiFE can prevent falls and increase physical function in older people with vision impairment, compared with usual care. Usual care refers to typical programs delivered by O&M Specialists to people with vision impairment. These programs focus on teaching people with vision impairment how to identify where they are and where they want to go (orientation), as well as how to move safely and efficiently from one place to another (mobility). Examples of a program could be orientation and mobility in the home, or catching public transport to work. As each program is specific to the goals of each person, they can vary dramatically in complexity, but are typically achieved within 6 months.

A process evaluation is particularly important in trials of complex interventions and is a common method in determining the factors which influence the level of success of an intervention [10]. The UK Medical Research Council (MRC) [10] recommend process evaluation protocols are published to ensure transparency in the development and implementation of interventions, as well as dissemination of results. This process evaluation protocol is designed to explore the development, implementation and efficacy of an intervention designed to improve physical function, and ultimately, reduce falls in older adults with vision impairment.

## Methods

In this paper, we outline the aims and corresponding methods for the PlaTFORM process evaluation. The overarching aim is to understand the relationship between program implementation and acceptability, and program outcomes [11]. More specifically, (1) to explore if the PlaTFORM trial was delivered as intended (i.e. idelity and dose delivered) and (2) to understand whether, how and why the intervention had an impact, through exploring participants’ perspectives and enactment of the intervention (i.e. participant receipt and participant enactment).

We aim to recruit 588 community dwelling people with vision impairment, aged 50 years or above. After baseline assessment, participants will be randomly assigned to the intervention or control group. The intervention group will receive the v-LiFE program plus usual care (an Orientation and Mobility program), while the control group will receive only usual care. This process evaluation will only include data from the intervention group.

### Program description

The v-LiFE program is designed to incorporate strength and balance activities into the everyday activities of older people with vision loss, living in the community, who are at high risk of falls. The intervention group will receive usual care as well the v-LiFE program over five weekly sessions, plus two booster visits and two phone calls within three months. If required, participants will receive up to two additional sessions. All sessions will be delivered in participants’ homes by O&M Specialists from Guide Dogs NSW/ACT. The control group will receive usual care.

The strength and balance activities will be catered to each participants’ physical function as they move through the program. As participants improve their strength and balance they will be encouraged to upgrade to more challenging exercises (i.e. reducing their base of support or increasing the weight lifted). O&M Specialists will be able to tailor their approach for each participant, which is in line with consensus that programs need to account for the individual context when delivering interventions [12]. Specially designed v-LiFE Participant manuals were produced for this population in audio, large print and electronic format.

### Implementation of v-LiFE

The implementation of the v-LiFE program will consist of three essential components [11]: training, intervention delivery and intervention enactment. Participants will also undergo a fourth component, assessment [11], at baseline and 12-months. These data will be collected as part of the trial outcomes for PlaTFORM and are described elsewhere [9].

#### The training component

More than 50 O&M Specialists from 10 offices, in six regions of NSW/ACT, will participate in two days of training. To assist in training and participation of those in regional areas, O&M Specialists will be able to take part in one of three possible training sessions (two will take place in Sydney NSW, and one in Northern NSW). The first day of training will consist of training in the delivery of the v-LiFE program and the second day will consist of training in research procedures. Each session will be facilitated by the same trainers (v-LiFE program: LC, JM).

#### The intervention delivery component

O&M Specialists will then deliver the v-LiFE program to older adults with vision impairment. This will be completed through (a) five weekly sessions, (b) two booster visits within three months, (c) two support calls after the visits are completed, and (d) if required, two additional sessions. Trainer and participant v-LiFE manuals have been designed for this population, which are to be closely followed in each session. To enhance self-efficacy, O&M Specialists are required to give positive reinforcement, encourage the use of self-monitoring tools and to assist in setting personal goals for short-term achievements.

#### The intervention enactment component

Participants will be required to incorporate the strength and balance activities, learnt during sessions, into their daily activities.

### Conceptualising v-LiFE with the behaviour change wheel

Michie et al. [13] developed the Behaviour Change Wheel (BCW), as a model for designing and evaluating interventions, as well as dissemination of results. The three domains of the BCW highlight the relationship of policy, intervention and the individual, which influence successful behaviour change. By participating in the v-LiFE program, participants will have to actively change their behaviour to incorporate the strength and balance activities into daily life. The BCW describes behaviour change through three *sources of behaviour* (inner wheel): capability (psychological, physical), opportunity (social, physical) and motivation (automatic, reflective). These behaviour changes will be facilitated by the O&M Specialists, which fall under *intervention functions* (middle wheel) of the BCW. Of the nine intervention functions, the components which are particularly relevant for our trial are: education, persuasion, coercion, training, incentivisation and enablement. Within the intervention functions are behaviour change techniques (BCTs), which are the ‘active ingredients’, designed to change behaviour. Guide Dogs NSW/ACT will support the O&M Specialists in their training of the v-LiFE program with participants. The BCW wheel includes seven *policy categories* (outer wheel), of which, service provision is considered particularly relevant to this study.

Table 1 shows specific measures that will be undertaken as part of the process evaluation and how they relate to the BCW model and BCTs.

**Table 1:** Process evaluation procedures mapped to measures, outcomes and Behaviour Change Techniques

Component		Question	Method/Measure	Data Type	Outcomes	^a^Behaviour Change Techniques (Intervention Function)
**1.**	Program Adherence (Fidelity)	How well did the O&M Specialists deliver the v-LiFE program to participants?	• A trained observer will observe 1–2 participant sessions with each instructor using a checklist, while making comments (20% of all v-LiFE program participants).	Quantitative	Higher scores indicate well delivered v-LiFE programs	Feedback on behaviour (Education/Persuasion/Coercion); Credible source (Persuasion); Instruction on how to perform the behaviour (Training); Problem solving (Enablement)
				Qualitative	Analysis of observer’s comments using the BCW as a framework	
**2.**	Complete Delivery (Dose delivered)	To what extent were all of the intended components of the v-LiFE program delivered to participants?	• O&M Specialists to record each time they complete a session, booster session, additional sessions and/or phone calls.	Quantitative	Higher scores indicate participants received a higher program dose	Self-monitoring of behaviour (Education/Incentivisation); Monitoring of behaviour by others without evidence of feedback (Coercion); Prompt/cues (Education)
**3.**	Participant Receipt (Dose received)	To what extent were participants engaged/satisfied with the v-LiFE program?	• Semi-structured interview (30 participants).	Qualitative	Analysis of transcripts using the BCW as a framework	Information about health consequences, self-monitoring of behaviour (Education); Monitoring of behaviour by others without evidence of feedback (Coercion)
			• Short answer survey (all participants).	Quantitative	Higher scores on the AFRIS indicate higher engagement to v-LiFE	
**4.**	Participant Enactment	To what extent were the participants completing the prescribed activities?	• The Self-Report Habit Index (post-program)	Quantitative	Higher scores on the Index indicate stronger habit formation	Self-monitoring of behaviour (Education/Incentivisation/Coercion); Feedback on behaviour (Education/Incentivisation/Coercion); Monitoring of outcome of behaviour by others without evidence of feedback (Incentivisation/Coercion

^a^ Michie et al. [13], BCW: Behaviour Change Wheel. AFRIS: Attitudes to Falls-Related Interventions Scale

### Process measures

The process evaluation is a mixed methods design. This includes quantitative (fidelity checklists, number of completed sessions and survey data), as well as qualitative (semi-structured interviews) data collection. Process evaluation measures include program adherence (fidelity), complete delivery (dose delivered), participant receipt (dose received) and participant enactment.

#### Program adherence

Program adherence (fidelity) is a measure of what extent the intervention was developed and used as intended [11]. The theoretical underpinning of v-LiFE is habit retraining [14]. As there are more than 50 O&M Specialists delivering the program, it is important to assess whether all the participants are receiving equivalent training. O&M Specialists need to encourage the participants to incorporate the skills they learn through sessions into everyday activities. This self-efficacy is facilitated through: positive reinforcement from the instructor, self-monitoring and setting achievable short-term goals.

Program adherence will be measured through direct observation of participant sessions by an observer trained in the delivery of v-LiFE. Each instructor will be observed completing 1–2 sessions with participants, to cover approximately 20% of all v-LiFE program participants. A v-LiFE fidelity tool checklist (Additional file 1: Appendix 1) will be used in each observation to ensure consistency in assessment. The checklist is designed to assess whether sessions adhere to the program as designed and set out in the program manual, and the quality of delivery, including: content, key features, credibility, skill, language, verbal and active teaching strategies. Wherever possible, the observer will also provide feedback to the Instructor to improve service delivery.

#### Complete delivery

Complete delivery (dose delivered) is a measure of the number of intended v-LiFE sessions and phone calls provided to participants [11]. In order for complete delivery, O&M Specialists will be required to deliver 5 weekly sessions, 2 booster visits and 2 follow-up phone calls. O&M Specialists are also able to provide an additional 2 sessions if required by the participants. Program session data will be collected from the Guide Dogs Customer Relationship Manager (CRM) database. As with any O&M program and associated program sessions, O&M Specialists will be required to detail the name, date, travel time, travel distance, session objectives and session outcomes in Guide Dogs CRM database. This will be a record of sessions and phone calls and personnel involved.

#### Participant receipt

Participant receipt (dose received) is a measure of participant’s engagement and understanding of the program content of v-LiFE, as well as their satisfaction with the program [11]. Participant receipt will be measured through semi-structured interviews and survey questions (Additional file 2: Appendix 2) following program completion. Participants in the intervention arm will be invited to complete the semi-structured interview and survey questions over the phone by a researcher not involved in the delivery of v-LiFE. Both the semi-structured and survey questions have been adapted from the Attitude to Falls-Related Intervention Scale (AFRIS). Based on the Theory of Planned Behaviour, the AFRIS helps to identify why or why not participants have engaged in a fall prevention intervention, through analysing what is considered acceptable to a participant [15].

#### Participant enactment

Participant enactment is a measure of what extent the knowledge and skills gained from completing the v-LiFE program are applied to participants’ everyday life. The v-LiFE program requires participants to actively engage in activities and opportunities that incorporate strength and balance into their daily routines. Habit formation is considered a core component of enabling people to perform LiFE activities routinely [14]. Consequently, in the initial v-LiFE session, the O&M Specialist will work with the participant on identifying opportunities in their daily routines which are associated with particular situations which will prompt the participant to practise the strength and balance activities. Participant enactment will be measured through the Self-Report Habit Index [16]. Hackney et al. [16] designed the index to measure habit strength, without the need to measure behavioural frequency of an activity. Habit strength refers to the frequent and satisfactory pairing of specific cues and automatic responses. For example, the pairing of a daily activity (specific cue), such as brushing teeth, with a v-LiFE activity (automatic response), such as practicing a tandem stand. Following the completion of the v-LiFE training, participants will be asked to complete the Self-Report Habit Index with a research assistant. The participant will be asked to answer the index for (a) a v-LiFE activity they enjoy or did not find challenging to incorporate into daily life, and (b) a v-LiFE activity they did not enjoy or found challenging to incorporate into daily life (Additional file 3: Appendix 3).

### Data analysis

**Fig. 1:**
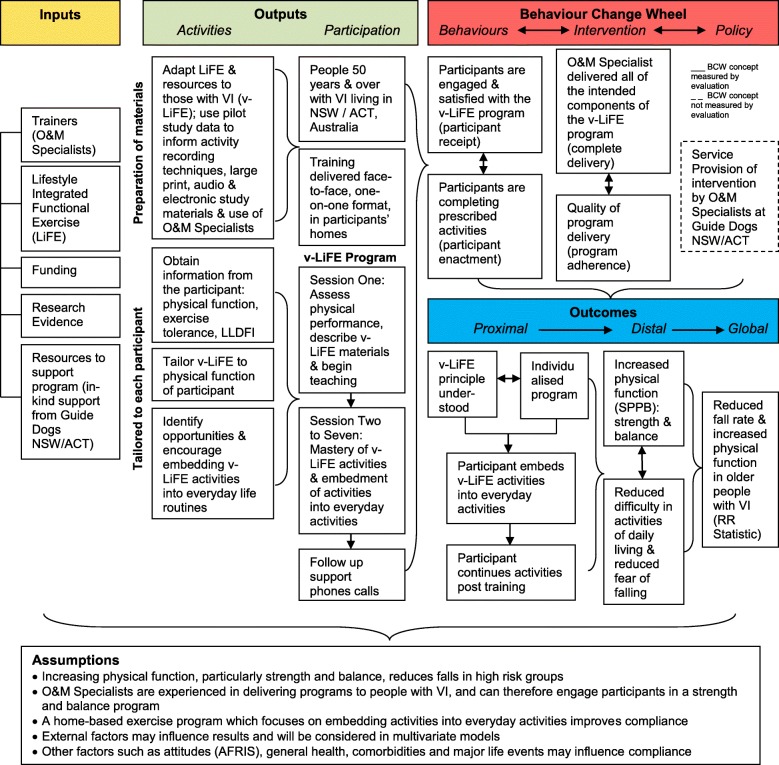
Logic model for PlaTFORM and v-LiFE implementation evaluation. O&M, Orientation and Mobility; VI, Vision Impairment; SPPB, Short Physical Performance Battery; LLFDI, Late Life Function Disability Index; RR Statistic, Risk Ratio Statistic; AFRIS, Attitudes to Falls Related Intervention Scale

Descriptive statistics will be used to summarise baseline demographic, vision and functional characteristics of intervention participants. In line with the MRC [10], quantitative data will be analysed prior to knowledge of trial outcomes to reduce bias. The quantitative and qualitative analyses will build upon one another, to help explain findings [10], and test the proposed logic model (Fig. 1).

#### Sample size

The PlaTFORM study was powered to be able to compare fall rates between control and intervention arms of the main RCT and required a total of 588 participants (294 per group) to detect a 30% between-group difference in falls rate, with 90% power at the 5% level, allowing for a 15% drop-out [9]. Consequently, quantitative data from up to 294 participants will be available for the process evaluation. This sample size will be sufficient to quantify the variation in scores for program fidelity, dose delivered, dose received through the AFRIS scale and enactment through the habit formation index. For qualitative data, it is expected this sample size will enable thematic saturation.

#### Quantitative data

Quantitative data will be collected through a number of sources, including: checklists, completion of program sessions and survey answers. Descriptive statistics of process measures will be summarised [10]. A correlation matrix (ranked) will determine any relationships between the process measures.

*Program adherence (fidelity)* will be measured through the score attained in the fidelity tool checklist as well as the variability in score attained in the domains within the fidelity checklist (Additional file 1: Appendix 1). For the initial session, there are 39 program delivery components the O&M Specialist will be scored on, on a scale of 1 to 3, where 1 = not done, 2 = could improve, 3 = well delivered (range 39 to 117). These components are separated into the domains: introduction, v-LiFE assessment tool, key points of the program explained, balance principles, strength principles, teaching the activity, planning and recording, and wrap up. For sessions two to six, there are 29 program delivery components the O&M Specialist will be scored on (range 29 to 87), using the same three point scoring method above. These components are separated into the domains: review of activities since last visit, teaching the activities, v-LiFE principles of balance and strength training reinforced, key points of program reinforced, safety reinforces, planning and recording, and wrap up at the end of the session.

*Complete delivery (dose delivered)* will be measured through number of v-LiFE sessions and phone calls delivered to participants by O&M Specialists, as a continuous variable (range 0 to 11). Program session data will be extrapolated from Guide Dogs CRM database.

*Participant receipt (dose received)* will be measured using AFRIS. This will be measured as a continuous variable (range 6 to 42) where higher scores indicate greater engagement in v-LiFE [15].

*Participant enactment* will be measured using the Self-Report Habit Index as a continuous variable (range 12 to 60). A score of 36 or higher indicates the presence of a habit. Lower scores indicate the absence of a habit [17].

#### Qualitative data

Qualitative data can assist in understanding the effects of interventions on behaviour change in complex randomised controlled trails [18]. The semi-structured interview transcripts will be coded within the framework of the Behaviour Change Wheel and BCTs [13]. More specifically, individual behaviour change (dose received and participant enactment) will be analysed using the behaviour sources of capability, opportunity and motivation. The acceptability of v-LIFE will be investigated with respect to the intervention functions and their associated BCTs. The aim is to identify, through report of the participants’ experience, why or why not behaviour change has occurred, as well as the acceptability of the v-LiFE program and its delivery by O&M Specialists.

## Discussion

A detailed protocol has been developed for a process evaluation nested within a randomised controlled trial of a home-based strength and balance program aimed at improving physical function, and ultimately, reducing falls in older people with vision impairment. The process evaluation will explore the implementation of the v-LiFE program in older people with vision impairment through investigations of adherence, delivery, receipt and enactment.

Ultimately, the findings of the process evaluation will guide the ongoing provision of fall prevention programs for older people with vision impairment. O&M Specialists provide individual programs to enhance the independence and safe mobility of people with vision impairment. However, at this stage, there are no specific programs being delivered to this high risk population, with the aim of improving physical function and reducing falls. This is a unique opportunity to understand the real world impact of program implementation on program outcomes, and translate these findings directly into practice. Garnering the views of participants and what they consider to be acceptable can assist to further refine the program and make it work better for this population. Such an understanding can have further implications for fall prevention of people in other high-risk populations.

There are no fall prevention programs based on balance and strength training which have been found to be effective for older people with vision impairment [19]. Understanding the mechanisms of impact can help us appreciate whether the findings of PlaTFORM are a result of program implementation, or the v-LiFE program itself. These results will also have relevance to policy makers looking to maximise the health and quality of life of people with vision impairment as they age.

### Strengths and limitations

Over 50 O&M Specialists will deliver the v-LiFE program to participants, so there is the potential of inconsistencies along service delivery. However, O&M Specialists will receive two days of training in delivery of the v-LiFE program by the same trainers, and will receive the same training materials and ongoing support. The fidelity tool (program adherence) will capture how well the Specialists deliver the v-LiFE program to participants. The large number of Specialists delivering the program allows for a wider reach of the program and reflects real world conditions. Another limitation is that the participants themselves will predominantly be recruited from the Guide Dogs NSW/ACT database and may have already undergone O&M training. As a consequence, these participants may already be quite pro-active, compared to those who may not have accessed rehabilitation services in the past. Thus, participants may already be used to ongoing weekly O&M sessions and be more receptive to this program than someone who has not accessed services.

To our knowledge, this will be the first time a process evaluation will be embedded in a randomised controlled trail that looks at strength and balance and falls in older people with vision impairment. The process evaluation will be collecting both qualitative and quantitative data in a mixed-method design, allowing for a holistic understanding of participants engagement with the v-LiFE program.

## Additional files

Additional file 1: **Appendix 1.** Program Adherence (fidelity). v-LiFE fidelity tool checklist, used in each observation of Orientation and Mobility Specialists to ensure consistency in delivery. (DOCX 17 kb)

Additional file 2: **Appendix 2.** Participant Receipt. Semi-structured interviews and survey questions, adapted from the Attitude to Falls-Related Intervention Scale (AFRIS). (DOCX 13 kb)

Additional file 3: **Appendix 3.** Participant Enactment. Survey questions, adapted from the Self-Report Habit Index. (DOCX 13 kb)

## Abbreviations

O&M: Orientation and Mobility
PlaTFORM: PrevenTing Falls in a high risk vision impaired population through specialist ORientation and Mobility services
v-LiFE: The Lifestyle Integrated Function Exercise (LiFE) program, adapted for older people with vision impairment
