# Supplementary material for: Understanding the implementation and efficacy of a home-based strength and balance fall prevention intervention in people aged 50 years or over with vision impairment: a process evaluation protocol
Source: BMC Health Serv Res. 2018 Jul 3;18:512. doi: 10.1186/s12913-018-3304-6 (PMC6029014; doi:10.1186/s12913-018-3304-6)
Supplement: Supplementary file 1 — Appendix 1. Program Adherence (fidelity). v-LiFE fidelity tool checklist, used in each observation of Orientation and Mobility Specialists to ensure consistency in delivery. (DOCX 17 kb) [file 12913_2018_3304_MOESM1_ESM.docx]

**Appendix 1 – Program Adherence (fidelity)**

| **v-LiFE Coaching Tool: Initial Session**  **Key: 0=Not done; 1=Could improve; 2=Well delivered; N/A** | | | |
| --- | --- | --- | --- |
| O&M Specialist (Trainer):  Participant (optional): | Coach (Observer): | | |
| **KEY ELEMENT** | | **RATING** | **COMMENTS** |
| **1. Introduction** | | | |
| Purpose and aims of program explained:  reduce falls / embed activities | | **0 1 2 N/A** |  |
| **2. v-LiFE Assessment Tool (vLAT)** | | | |
| Tool completed | | **0 1 2 N/A** |  |
| Principles introduced during assessment | | **0 1 2 N/A** |  |
| **3. Key Points of Program explained** | | | |
| Embed activities | |  |  |
| Change habits | |  |  |
| Practice | |  |  |
| Build in prompts / reminders / situational or environmental cues to prompt doing the activity | |  |  |
| Participant will Look for opportunities | |  |  |
| Modify environment to increase challenge | |  |  |
| Challenging yourself (no set number) | |  |  |
| Safety | |  |  |
| Key points reinforced throughout the session | |  |  |
| Overall | | **0 1 2 N/A** |  |
| **4. BALANCE PRINCIPLES** | | | |
| **Explains concept of Challenge your Balance** | | **0 1 2 N/A** |  |
| **Explains balance principles:** | |  |  |
| Reduce base of support | | **0 1 2 N/A** |  |
| Shift weight / move to limits of stability | | **0 1 2 N/A** |  |
| Stepping over objects | | **0 1 2 N/A** |  |
| Related balance principles to improving function and / or preventing falls | | **0 1 2 N/A** |  |
| **5. STRENGTH PRINCIPLES** | | | |
| **Explains concept of Load your muscles** | | **0 1 2 N/A** |  |
| **Load your muscles by:** | |  |  |
| Increasing times you use | | **0 1 2 N/A** |  |
| Move slowly | | **0 1 2 N/A** |  |
| Use fewer muscles | | **0 1 2 N/A** |  |
| Increase the weight | | **0 1 2 N/A** |  |
| Related strength principles to improving function and / or preventing falls | | **0 1 2 N/A** |  |
| **6. Teaching the Activity** | | | |
| Trainer and participant decide on activities to perform | | **0 1 2 N/A** |  |
| Trainer demonstrates the v-LiFE activities in the appropriate situation | | **0 1 2 N/A** |  |
| Participant performs activity in situation identified | | **0 1 2 N/A** |  |
| Participant technique corrected as needed | | **0 1 2 N/A** |  |
| Appropriate number of activities taught for participant’s ability | | **0 1 2 N/A** |  |
| Appropriate level of activities taught for participant’s ability | | **0 1 2 N/A** |  |
| Activities related to principles | | **0 1 2 N/A** |  |
| Trainer refers to **Participants Manual** (if appropriate) to explain and reinforce the activities taught | | **0 1 2 N/A** |  |
| **7. Planning and Recording** | | | |
| **Daily Routine Chart** (or modified tool) used to plan how, when, where activity will be performed and embedded | | **0 1 2 N/A** |  |
| **Activity Planner** (or modified tool) - purpose and use explained | | **0 1 2 N/A** |  |
| **Activity Planner** (or modified tool) used to record plan for activity performance | | **0 1 2 N/A** |  |
| **Activity Counter** (or modified tool) - purpose and use explained | | **0 1 2 N/A** |  |
| Activities to be counted on **Activity Counter** identified | | **0 1 2 N/A** |  |
| Recording of activities to be counted and days for counting done | | **0 1 2 N/A** |  |
| **8. Wrap up – at the end of the session** | | | |
| Trainer explains / reinforces what is required of participant until next session  Activity performance  Recording  Read manual | | **0 1 2 N/A** |  |
| Trainer has participant explain what they are required to do until the next session  Corrects and clarifies as needed | | **0 1 2 N/A** |  |
| **9. Trainer review** | |  |  |
| Was there sufficient time to deliver the session to a high fidelity | | **Yes / No** |  |

**Coach Only:**

| **10. Trainer Feedback** | |
| --- | --- |
| If given, was the trainer open to and actively motivated to improve their delivery based on feedback? |  |

| **v-LiFE Coaching Tool: Sessions 2 – 6**  **Key: 0=Not done; 1=Could improve; 2=Well delivered; N/A** | | |
| --- | --- | --- |
| Session (circle): 2 3 4 5 6 | Participant (optional): | |
| O&M Specialist (Trainer): | Coach (Observer): | |
| **KEY ELEMENT** | **RATING** | **COMMENTS** |
| **1. Review of activities since last visit** | | |
| Recording sheets checked and problems identified | **0 1 2 N/A** |  |
| Review previous activities to identify any problems | **0 1 2 N/A** |  |
| Technique corrected if required | **0 1 2 N/A** |  |
| Trainer encourages / facilitates participant to engage in problem solving | **0 1 2 N/A** |  |
| Participant & Trainer identify appropriate upgrade to previous activities (if appropriate) | **0 1 2 N/A** |  |
| **2. Teaching the Activities** | | |
| Participant and trainer identify appropriate NEW activities to teach using DRC as appropriate | **0 1 2 N/A** |  |
| Trainer demonstrates the new v-LiFE activities in the appropriate situation | **0 1 2 N/A** |  |
| Participant performs new activity in situation identified & technique corrected as needed | **0 1 2 N/A** |  |
| Appropriate level of activities taught for participant’s ability | **0 1 2 N/A** |  |
| Appropriate number of activities taught for participants ability | **0 1 2 N/A** |  |
| Trainer refers to **Participants Manual** (if appropriate) to explain and reinforce the activities taught | **0 1 2 N/A** |  |
| **3. v-LiFE principles of balance and strength training reinforced** | | |
| Challenge your balance | **0 1 2 N/A** |  |
| Load your muscles | **0 1 2 N/A** |  |
| Trainer relates the principles and activities to function and / or preventing falls | **0 1 2 N/A** |  |
| **4. Key Points of Program reinforced** | | |
| Embed activities | **0 1 2 N/A** |  |
| Change habits | **0 1 2 N/A** |  |
| Build in situational or environmental prompts or reminders as cues to activity performance | **0 1 2 N/A** |  |
| Look for opportunities | **0 1 2 N/A** |  |
| Challenging yourself (no set number) | **0 1 2 N/A** |  |
| Modify environment to increase challenge | **0 1 2 N/A** |  |
| Continuous upgrading | **0 1 2 N/A** |  |
| **5. Safety Reinforced** | | |
| Verbally & by actions or demonstration | **0 1 2 N/A** |  |
| **6. Planning and Recording** | | |
| Trainer encourages participant to be engaged in planning how, when and where the activities will be embedded | **0 1 2 N/A** |  |
| **Activity Planner** (or modified tool) used to record plan for activity performance. Upgrades included | **0 1 2 N/A** |  |
| Activities to be counted on **Activity Counter** identified | **0 1 2 N/A** |  |
| Recording of activities to be counted and days for counting done | **0 1 2 N/A** |  |
| Participant is generating their own ideas of activities that can be incorporated into daily activities (by sessions 6) | **0 1 2 N/A** |  |
| **7. Wrap Up at the end of the session** | | |
| Trainer explains / reinforces what is required of participant until next session  Performance of activities  Recording | **0 1 2 N/A** |  |
| Trainer has participant explain what they are required to do until the next session  Corrects and clarifies as needed | **0 1 2 N/A** |  |
| **8. Trainer review** | | |
| Was there sufficient time to deliver the session to a high fidelity | **Yes / No** |  |

**Coach Only:**

| **10. Trainer Feedback** | |
| --- | --- |
| If given, was the trainer open to and actively motivated to improve their delivery based on feedback? |  |
