# Supplementary material for: Understanding the implementation and efficacy of a home-based strength and balance fall prevention intervention in people aged 50 years or over with vision impairment: a process evaluation protocol
Source: BMC Health Serv Res. 2018 Jul 3;18:512. doi: 10.1186/s12913-018-3304-6 (PMC6029014; doi:10.1186/s12913-018-3304-6)
Supplement: Supplementary file 2 — Appendix 2. Participant Receipt. Semi-structured interviews and survey questions, adapted from the Attitude to Falls-Related Intervention Scale (AFRIS). (DOCX 13 kb) [file 12913_2018_3304_MOESM2_ESM.docx]

**Appendix 2 – Participant Receipt**

Both the semi-structured interviews and survey questions have been adapted from the Attitude to Falls-Related Intervention Scale (AFRIS). The semi-structured interview questions are designed to elicit longer and more in depth answers to be used for qualitative data, whereas, the survey questions are designed to elicit short answers that can be used as quantitative data.

**Semi-structured Interview Questions**

1. Did you think the v-LiFE program was good for you? How?
2. Do you feel your balance and strength has changed? How?
3. Were there any unexpected outcomes from doing the v-LiFE program?
4. What is the main thing you learned from the lessons?
5. Has v-LiFE impacted on your everyday activities? How?
6. Describe how you found doing the v-LiFE program? easy/hard?, enjoyable?
7. Do you find that your mobility has changed since doing the activities of the v-LiFE program?
8. How do you feel about continuing to do the v-LiFE program? Are there any things preventing you?
9. Would you recommend the v-LiFE program to others like yourself?

**Survey questions**

*‘We want to know whether or not you think v-LiFE was right for YOU, and the reasons why it was or was not right for you* (coach about scale)*.’*

| **Questions** | | **Disagree strongly** | **Disagree** | **Disagree slightly** | **Neither agree nor disagree** | **Agree slightly** | **Agree** | **Agree strongly** |
| --- | --- | --- | --- | --- | --- | --- | --- | --- |
| **1.** | Doing v-LiFE was good for me |  |  |  |  |  |  |  |
| **2.** | Doing v-LiFE made me feel confident |  |  |  |  |  |  |  |
| **3.** | Other people whose opinions matter to me (e.g. family, friends, doctor) think it was a good idea for me to do v-Life |  |  |  |  |  |  |  |
| **4.** | If I want to, it is easy for me to do v-LiFE activities |  |  |  |  |  |  |  |
| **5.** | I am the kind of person who suits v-LiFE |  |  |  |  |  |  |  |
| **6.** | I intend to do v-LiFE activities |  |  |  |  |  |  |  |
