# Supplementary material for: Understanding the implementation and efficacy of a home-based strength and balance fall prevention intervention in people aged 50 years or over with vision impairment: a process evaluation protocol
Source: BMC Health Serv Res. 2018 Jul 3;18:512. doi: 10.1186/s12913-018-3304-6 (PMC6029014; doi:10.1186/s12913-018-3304-6)
Supplement: Supplementary file 3 — Appendix 3. Participant Enactment. Survey questions, adapted from the Self-Report Habit Index. (DOCX 13 kb) [file 12913_2018_3304_MOESM3_ESM.docx]

**Appendix 3 – Participant Enactment**

**The Self-Report Habit Index**

*‘What activity have you been implementing, or trying to implement routinely?’* _______________________

| **Questions** | **Very much agree** | **Somewhat agree** | **Neutral** | **Somewhat disagree** | **Very much disagree** |
| --- | --- | --- | --- | --- | --- |
| 1. I do this v-LiFE activity frequently. |  |  |  |  |  |
| 2. I do this v-LiFE activity automatically. |  |  |  |  |  |
| 3. I do it without having to consciously remember. |  |  |  |  |  |
| 4. It makes me feel weird if I do not do it. |  |  |  |  |  |
| 5. I do it without thinking |  |  |  |  |  |
| 6. It would require effort not to do it |  |  |  |  |  |
| 7. It belongs to my (daily, weekly, monthly) routine |  |  |  |  |  |
| 8. I start doing it before I realise I'm doing it. |  |  |  |  |  |
| 9. I would find it hard not to do. |  |  |  |  |  |
| 10. I have no need to think about doing it. |  |  |  |  |  |
| 11. I think of the activity as something that's typically "me." |  |  |  |  |  |
| 12. I have been doing it now for a long time. |  |  |  |  |  |

*‘What reminds you or prompts you to do the activity?’* Specify cue: _________________________________

*‘Did it work well?’_________________________________________________________________________*

Type of cue: time / visual / activity / other (specify): _____________________________________________

Repeat above with an activity that has been challenging for the participant to implement, or maintain.
